# Impact of heat stress on prolactin-mediated ovarian JAK-STAT signaling in postpubertal gilts

**DOI:** 10.1093/jas/skac118

**Published:** 2022-07-01

**Authors:** Crystal M Roach, Katie L Bidne, Matthew R Romoser, Jason W Ross, Lance H Baumgard, Aileen F Keating

**Affiliations:** Department of Animal Science, Iowa State University, Ames, IA 50011, USA; Department of Animal Science, Iowa State University, Ames, IA 50011, USA; Department of Animal Science, Iowa State University, Ames, IA 50011, USA; Department of Animal Science, Iowa State University, Ames, IA 50011, USA; Department of Animal Science, Iowa State University, Ames, IA 50011, USA; Department of Animal Science, Iowa State University, Ames, IA 50011, USA

**Keywords:** gilts, heat stress, JAK-STAT, ovary, prolactin, seasonal infertility

## Abstract

Heat stress (**HS**) compromises almost every aspect of animal agriculture including reproduction. In pigs, this infecundity is referred to as seasonal infertility (**SI**), a phenotype including ovarian dysfunction. In multiple species, HS-induced hyperprolactinemia has been described; hence, our study objectives were to characterize and compare HS effects on circulating prolactin (**PRL**) and ovarian Janus kinase/signal transducer and activator of transcription (**JAK-STAT**) signaling during the follicular (**FOL**) or luteal (**LUT**) phases of the estrous cycle in postpubertal gilts. Gilts were estrus synchronized using altrenogest and environmental treatments began immediately after altrenogest withdrawal. For the FOL study: postpubertal gilts were allocated to constant thermoneutral (**TN**; *n* = 6; 20 ± 1.2 °C) or cyclical HS (*n* = 6; 25 to 32 ± 1.2 °C) conditions for 5 d. In the LUT study: postpubertal gilts were assigned to either TN (*n* = 7; 20 ± 2.6 °C) or cyclical HS (*n* = 7; 32 to 35 ± 2.6 °C) conditions from 2 to 12 days postestrus (**dpe**). Blood was collected by jugular venipuncture for PRL quantification on day 5 in the FOL and on day 0 and day 12 in the LUT gilts. Ovaries and corpora lutea (**CL**) were obtained from euthanized FOL and LUT gilts on day 5 and day 12, respectively. Western blotting was performed to quantify prolactin receptor (**PRLR**) and JAK/STAT pathway protein abundance. In the FOL phase, no difference (*P* = 0.20) in circulating PRL between thermal groups was observed. There was no effect (*P* ≥ 0.34) of HS on PRLR, signal transducer and activator of transcription 3 (**STAT3**), signal transducer and activator of transcription 5α (**STAT5α**), and phosphorylated signal transducer and activator of transcription α/β tyrosine 694/699 (**pSTAT5α/β**^**Tyr694/699**^) abundance and Janus kinase 2 (**JAK2**), phosphorylated janus kinase 2 tyrosine 1007/1008 (**pJAK2**^**Tyr1007/1008**^), STAT1, phosphorylated signal transducer and activator of transcription 1 tyrosine 701 (**pSTAT1**^**Tyr701**^), phosphorylated signal transducer and activator of transcription 1 serine 727 (**pSTAT1**^**Ser727**^), and phosphorylated signal transducer and activator of transcription 3 tyrosine 705 (**pSTAT3**^**Tyr705**^) were undetectable in FOL gilt ovaries. Ovarian pSTAT5α/β^Tyr694/699^ abundance tended to moderately increase (4%; *P* = 0.07) in FOL gilts by HS. In the LUT phase, circulating PRL increased progressively from 2 to 12 dpe, but no thermal treatment-induced difference (*P* = 0.37) was noted. There was no effect (*P* ≥ 0.16) of HS on CL abundance of PRLR, pJAK2^Tyr1007/1008^, JAK2, STAT1, pSTAT1^Tyr701^, pSTAT1^Ser727^, pSTAT3^Tyr705^, STAT5α, or pSTAT5α/β^Tyr694/699^. In LUT phase, CL STAT3 abundance was increased (11%; *P* < 0.03) by HS. There was no impact of HS (*P* ≥ 0.76) on levels of pJAK2^Tyr1007/1008^ and pSTAT5α/β^Tyr694/699^ in LUT gilts; however, the CL pSTAT3^Tyr705^:STAT3 ratio tended to be decreased (*P* = 0.10) due to HS. These results indicate an HS-induced estrous cycle-stage-dependent effect on the ovarian JAK/STAT pathway, establishing a potential role for this signaling pathway as a potential contributor to SI.

## Teaser

Heat stress causes hyperprolactinemia in several species. This study examined a role for prolactin-mediated ovarian and luteal JAK-STAT alterations during heat stress in the pig.

## Introduction

Climate change, specifically heat stress (**HS**), negatively affects global animal agriculture productivity, and threatens food security (Baumgard et al., 2012; United Nations, 2020). HS occurs when environmental conditions (ambient temperature, relative humidity, and solar radiation) coupled with endogenous heat production generate a thermal condition culminating in increased core body temperature (Bernabucci et al., 2010). In the pig industry, HS-induced infecundity is referred to as seasonal infertility (**SI**) (Ross et al., 2017); which is manifested phenotypically as delayed puberty onset, prolonged weaning to estrus interval, reduced establishment of pregnancy, and early embryonic death (Love et al., 1993; Xue et al., 1994; Tast et al., 2002). HS modifies ovarian steroidogenic enzyme abundance (Nteeba et al., 2015), induces an ovarian heat shock protein response (Seibert et al., 2019), increases granulosa cell apoptotic signaling (Luo et al., 2016), and impairs oocyte quality and gonadotrophin production (Tseng et al., 2006). In addition, HS reduces follicular dominance following recruitment (Ozawa et al., 2005), increases ovarian autophagy (Hale et al., 2017), and reduces follicular gonadotropin receptor expression and steroid hormone production (Ozawa et al., 2005). Further, HS reduces corpora lutea weight and size in gilts (Bidne et al., 2019) and ovarian insulin receptor (**INSR**) messenger RNA (**mRNA**) abundance (Nteeba et al., 2015). Consequently, there are multiple avenues by which HS can compromise ovarian function and deleteriously influence swine reproduction.

Prolactin (**PRL**) is a polypeptide hormone produced and secreted by lactotrophs within the anterior pituitary gland (Riddle et al., 1933). PRL is inhibited by hypothalamic dopamine or stimulated by neuropeptides, dopamine antagonists, estrogen, and thyrotropin-releasing hormone (Kamberi et al., 1970; Ben-Jonathan, 1985). To elicit a cellular response, PRL binds to the prolactin receptor (**PRLR**), activating the janus kinase/signal transducer and activator of transcription (**JAK-STAT**), mitogen-activated protein kinase, or phosphatidylinositol-3 kinase cellular pathways. Janus kinase 2 (**JAK2**) dimerization and autophosphorylation recruits STAT 1, 3, and 5 proteins by initiating phosphorylation, dimerization, and nuclear translocation for transcription of PRL-responsive genes. The porcine PRLR gene is located on chromosome 16 (Vincent et al., 1997), and circulating PRL is increased by HS in multiple species including pigs (Baumgard and Rhoads, 2013). A genome-wide association study associated thermotolerance in pigs with gene variants on chromosome 16 (Kim et al., 2018). Furthermore, porcine *PRLR* gene variants are associated with an increased total number born and number born alive (Vincent et al., 1998). Ergo, PRL is ostensibly a key endocrine participator in SI.

In an effort to remain euthermic, HS animals reduce feed intake to minimize the thermic effect of digestion. Despite reduced nutrient consumption, circulating insulin is increased during HS and this is especially apparent when compared to thermal neutral animals on a similar plane of nutrition (Itoh et al., 1998; Wheelock, 2006; Sans-Fernandez et al., 2015). HS compromises gastrointestinal barrier integrity in multiple species including pigs (Pearce et al., 2012, 2013, 2014; Liu et al., 2016; Gabler et al., 2018) and this allows for the infiltration of luminal antigens (i.e., lipopolysaccharide [**LPS**]; Catron et al., 2019) into the local and systemic circulation (Pearce et al., 2013). Immune activation through acute injection of interleukin-1 beta, a pro-inflammatory cytokine induced by LPS, increased circulating insulin in mice (Dror et al., 2017) demonstrating a role for inflammatory mediators in metabolism homeostasis and the response to an immune challenge. LPS-induced activation of inflammatory cytokines and Toll-like receptor 4/nuclear factor-kappa B signaling is blunted by PRL in vitro (Olmos-Ortiz et al., 2019), suggesting an anti-inflammatory role of PRL. Heat-induced hyperprolactinemia occurs in humans (Iguchi et al., 2012), ruminants (Smith et al., 1977; Schillo et al., 1978; Ronchi et al., 2001; Alamer, 2011), and nonruminant (Baumgard and Rhoads, 2013) livestock species. The precise mechanism by which HS increases PRL is unknown; however, the PRL response may be involved with water metabolism (Collier et al., 1982), pelage molting (Foitzik et al., 2009), and/or heat shock protein induction (Blake et al., 1995). A thermal role for PRL is supported by its function in sweat production (Kaufman and Mackay, 1983), intracellular and extracellular osmotic fluid balance (Kaufman and Mackay, 1983), and water intake (Schams and Himmler, 1978; Schams et al., 1980). Interestingly, PRL also functions as a growth factor for pancreatic beta cells via the JAK/STAT pathway (Nielsen et al., 1992) and induces glucose-stimulated insulin secretion and insulin production in rat islet beta cells during fetal (Møldrup et al., 1993; Freemark et al., 1995; Royster et al., 1995), neonatal (Brelje and Sorenson, 1991; Brelje et al., 1994), and adult development (Møldrup et al., 1993), suggesting PRL may partially mediate hyperinsulinemia during HS (Baumgard and Rhoads, 2013).

Little is known whether HS increases circulating PRL and impacts porcine ovarian function via the JAK-STAT pathway, although studies have discovered HS-induced changes in PRL levels in pregnant (Li et al., 2021) and nonpregnant (Zhang et al., 2019) sows. Our previous discoveries on ovarian impacts of HS-induced hyperinsulinemia and a documented relationship between insulin and PRL provided the rationale for our supposition. The study objectives were 3-fold: to determine HS effects on circulating PRL, to examine if ovarian PRL-induced JAK/STAT protein abundance is affected during HS, and to determine the influence of estrous cycle phase on JAK/STAT signaling during HS in postpubertal gilts. We hypothesized that HS would impact circulating PRL and alter ovarian JAK/STAT pathway proteins during the FOL and LUT phases.

## Materials and Methods

### Animals and tissue collection

The Institutional Animal Care and Use Committee at Iowa State University approved all animal procedures. This study utilized tissues collected from previously described experiments (Hale et al., 2017; Dickson et al., 2018; Bidne et al., 2019; Seibert et al., 2019; Romoser et al., 2022). Gilts were estrus synchronized with 15 mg/d of altrenogest (Matrix; Merck Animal Health, Summit, NJ), a synthetic progestogen, for 14 d under constant thermoneutral conditions (**TN**; 20 ± 2.6 °C). For the follicular phase (**FOL**) of the project, 12 postpubertal gilts (126.0 ± 21.6 kg) were allocated to TN (*n* = 6; 20 ± 1.2 °C) or cyclic HS conditions (*n* = 6; 25 to 32 ± 1.2 °C) for 5 d beginning immediately after altrenogest withdrawal (Hale et al., 2017; Dickson et al., 2018). This duration encompassed the entirety of the follicular phase of the estrous cycle and ovaries were collected before ovulation. For the luteal phase (**LUT**) of the project, 14 postpubertal gilts (167.0 ± 10 kg) were assigned to either TN (*n* = 7; 20 ± 2.6 °C) or diurnal HS (*n* = 7; 32-35 ± 2.6 °C) conditions from 2 to 12 days postestrus (**dpe**) (Bidne et al., 2019). Beginning 4 d post-altrenogest withdrawal, gilts were checked for behavioral signs of standing estrus twice daily using boar exposure and an animal was classified as in estrus when she would stand for back pressure. Rectal temperature and respiration rate were collected twice daily from each animal. At the end of the experimental period, all animals were euthanized by captive bolt followed by exsanguination. For FOL gilts, the whole ovary, and for LUT gilts, the corpora lutea (**CL**), were removed and flash-frozen in liquid nitrogen. Frozen samples were powdered on dry ice using a mortar and pestle and stored at −80 °C until further analysis.

### Blood collection

Blood samples were collected into sodium heparin tubes and centrifuged for 15 min at 1,500 × *g* at 4 °C for plasma collection. Plasma samples were collected at day 5 of the estrous cycle from FOL gilts and at day 0 and 12 dpe from LUT gilts as described in Dickson et al. (2018) and Bidne et al. (2019), respectively.

### PRL quantification

Porcine PRL in plasma for FOL and LUT gilts was measured using a double-antibody sandwich enzyme-linked immunosorbent assay kit (MBS777852, MyBioSource Inc., San Diego, CA). Intra- and interassay coefficient of variation was less than 10% and 15%, respectively. The standard curve range was between 1.0 and 40 ng/mL, with a sensitivity of 0.1 ng/mL. All procedures were according to the manufacturer’s instructions.

### Protein isolation and western blotting

Powdered ovarian (FOL) or CL (LUT) tissue was weighed and lysed by tissue lysis buffer (1% Trition X-100 [T-6878], 50 mM 4-(2-hydroxyethyl)-1-piperazineethanesulfonic acid [H3537], 150 mM sodium chloride [S3014], 10% glycerol [G5516], 50 mM sodium fluoride [S7920], 2 mM ethylenediaminetetraacetic acid [E7889], 1% sodium dodecyl sulfate [L3771], Sigma Aldrich, St. Louis, MO) supplemented with Halt protease and phosphatase inhibitor cocktail (P178442, Thermo Scientific, Rockford, IL). Lysed tissue was homogenized by sonication and incubated on ice for 30 min. Protein lysate was centrifuged at 10,000 rpm for 15 min at 4 °C and supernatant was removed and stored at −80 °C. Protein concentration was quantified using a Pierce bicinchoninic acid Protein Assay Kit (23227, Thermo Scientific) and spectrophotometry detection. Samples were prepared with 1× Laemilli dye (161-0737, Bio-Rad Laboratories, Hercules, CA), diethylpyrocarbonate-treated water (750024, Thermo Scientific), and incubated at 95 °C for 5 min. Protein (60 µg) was separated on 4% to 20% Mini-PROTEAN TGX Precast Protein Gels (4561096, Bio-Rad) by electrophoresis for 90 min at 110 V. Separated proteins were transferred to nitrocellulose membranes (iBlot 2 Transfer Stacks, Thermo Scientific) by iBlot 2 Dry Transfer System (Protocol 0: 20 V for 1 min, 23 V for 4 min, and 25 V for 2 min) for 7 min. Protein transfer was determined by incubation in 1× Ponceau S (BP103-10, Thermo Scientific) stain and the image was captured using the FOTO/Analyst Investigator (FOTODYNE Incorporated). Membranes were washed in phosphate-buffered saline (**PBS**) with 0.2% Tween-20 (**PBST**; PBS: BP665-1, Tween-20: BP337-500, Thermo Scientific) and blocked at room temperature in 5% nonfat dry milk with PBST. Membranes were incubated with primary antibodies directed against PRLR (MA1-610, Thermo Scientific), JAK2 (3230, Cell Signaling, Danvers, MA), phosphorylated janus kinase 2 tyrosine 1007/1008 (**pJAK2**^**Tyr1007/1008**^; 3771, Cell Signaling), signal transducer and activator of transcription 1 (**STAT1**; 14994, Cell Signaling), phosphorylated signal transducer and activator of transcription 1 serine 727 (**pSTAT1**^**Ser727**^; 9177, Cell Signaling), phosphorylated signal transducer and activator of transcription 1 tyrosine 701 (**pSTAT1**^**Tyr701**^; 9167, Cell Signaling), signal transducer and activator of transcription 3 (**STAT3**; 4904, Cell Signaling), phosphorylated signal transducer and activator of transcription 3 tyrosine 705 (**pSTAT3**^**Tyr705**^; 9131, Cell Signaling), signal transducer and activator of transcription 5α (**STAT5α**; 13-3600, Thermo Scientific), phosphorylated signal transducer and activator of transcription α/β tyrosine 694/699 (**pSTAT5α/β**^**Tyr694/699**^; sc-81524, Santa Cruz), and beta-actin (**ACTB**) (sc-47778, Santa Cruz; dilutions listed in Table 1) overnight at 4 °C for 16 h. Membranes were washed and incubated with goat anti-mouse immunoglobulin G (**IgG**)-horseradish peroxidase (**HRP**)-linked or goat anti-rabbit IgG-HRP-linked secondary antibodies (dilutions listed in Table 1) for 1 h at room temperature. Technical controls included western blots on FOL ovarian and LUT CL lysate with incubation in primary (no secondary) antibody and secondary (no primary) antibody to confirm specificity. Membranes were incubated in Signalfire enhanced chemiluminescence reagent (6683, Cell Signaling Technology, Danvers, MA) for 7 min and exposed to chemiluminescence x-ray film in a dark room. Proteins of interest were quantified using densitometry ImageJ software (NCBI). Ponceau S staining and ACTB were used as loading controls, and each protein measurement was normalized to ACTB levels.

**Table 1.** Antibodies and dilutions of proteins used in western blotting

Primary antibody	Description	Host	Primary dilution	Secondary dilution
PRLR	Prolactin receptor	Mouse	1:300	1:500
JAK2	Janus kinase 2	Rabbit	1:500	1:100
pJAK2^Tyr1007/1008^	Phospho-Jak 2	Rabbit	1:100	1:3,000
STAT1	Signal transducer and activator of transcription 1	Rabbit	1:500	1:1,000
pSTAT1^Ser727^	Phospho-Stat 1	Rabbit	1:500	1:1,000
pSTAT1^Tyr701^	Phospho-Stat 1	Rabbit	1:500	1:1,000
STAT3	Signal transducer and activator of transcription 3	Rabbit	1:250 to 1:1,000	1:500 to 1:1,000
pSTAT3^Tyr705^	Phospho-Stat 3	Rabbit	1:300	1:500
STAT5α	Signal transducer and activator of transcription 5 alpha	Mouse	1:250	1:500
pSTAT5α/β^Tyr694/699^	Phospho-Stat 5 alpha/beta	Mouse	1:300	1:500
ACTB	Beta-actin	Mouse	1:500	1:2,000

### Statistical analysis

Plasma PRL levels and protein abundance were compared by unpaired *t*-test with Welch’s correction, assuming unequal variances in GraphPad Prism 9. Data are presented as mean ± SEM. Statistical significance was considered at *P* < 0.05 and a trend for significance was considered if 0.05 ≥ *P* ≤ 0.10.

## Results

### Impact of HS on circulating plasma PRL level

**Figure 1.**
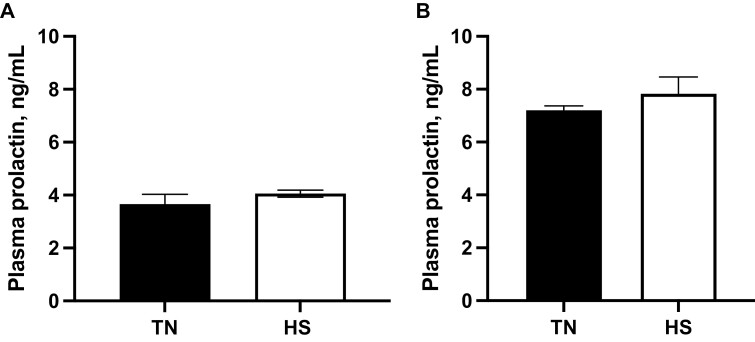
Impact of heat stress on circulating plasma PRL level. (A) Gilts were exposed to TN (20 ± 1.2 °C) or cyclic HS (25 to 32 ± 1.2 °C) during the follicular phase for 5 d. (B) Gilts were exposed to TN (20 ± 2.6 °C) or diurnal HS (32 to 35 ± 2.6 °C) during the luteal phase from 3 to 12 dpe. The concentration of circulating PRL (ng/mL) ± SEM is presented. Abbreviations: dpe, days postestrus; HS, heat stress; PRL, prolactin; TN, thermoneutral.

There was no impact (*P* = 0.20; Figure 1A) of HS on plasma PRL levels in FOL gilts on day 5. There was no effect of HS on circulating plasma PRL (*P* = 0.37; Figure 1B) on 12 dpe in LUT gilts.

### Effect of HS on PRLR protein

HS did not affect ovarian (*P* = 0.26) and luteal (*P* = 0.42) PRLR protein abundance in either the FOL or LUT phase, respectively (data not shown).

### Consequence of HS on JAK2 and pJAK2^Tyr1007/1008^ protein level

In FOL gilts, ovarian JAK2 and pJAK2^Tyr1007/1008^ protein was undetectable. In LUT gilts, luteal JAK2 protein was measurable, but there was no difference (*P* = 0.16) in luteal JAK2 protein abundance as a result of HS relative to TN controls (data not shown). Luteal protein abundance of pJAK2^Tyr1007/1008^ was unaltered (*P* = 0.88) by HS (data not shown).

### HS effect on STAT3 and pSTAT3^Tyr705^ protein abundance

**Figure 2.**
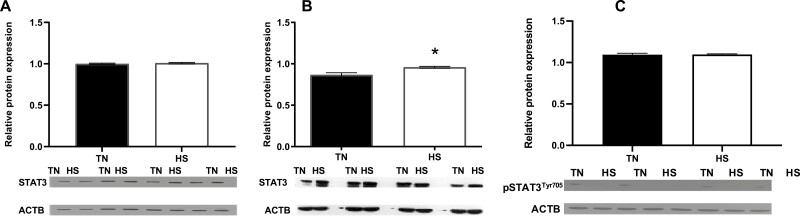
Heat stress effect on STAT3 and pSTAT3^Tyr705^. (A) Gilts were exposed to TN (20 ± 1.2 °C) or cyclic HS (25.4 to 32 ± 1.2 °C) during the follicular phase for 5 d. (B, C) Gilts were exposed to TN (20 ± 2.6 °C) or diurnal HS (32 to 35 ± 2.6 °C) during the luteal phase from 3 to 12 dpe. Western blots with positive staining for STAT3 or pSTAT3^Tyr705^ or ACTB proteins are presented. Bar charts represent the relative STAT3:ACTB or pSTAT3^Tyr705^:ACTB protein abundance ± SEM. *P < 0.05. Abbreviations: ACTB, beta-actin; dpe, days postestrus; HS, heat stress; pSTAT3^Tyr705^, phosphorylated signal transducer and activator of transcription 3 tyrosine 705; STAT3, signal transducer and activator of transcription 3; TN, thermoneutral

No difference (*P* = 0.55; Figure 2A) in STAT3 protein abundance was observed in FOL gilt ovaries. pSTAT3^Tyr705^ abundance was undetectable in FOL HS gilts. In LUT gilts, luteal STAT3 protein abundance was increased (*P* = 0.03; Figure 2B) due to HS. Luteal pSTAT3^Tyr705^ was unaltered (*P* = 0.96; Figure 2C) during HS relative to TN controls.

### HS impact on STAT5α and pSTAT5α/β^Tyr694/699^ ovarian protein

**Figure 3.**
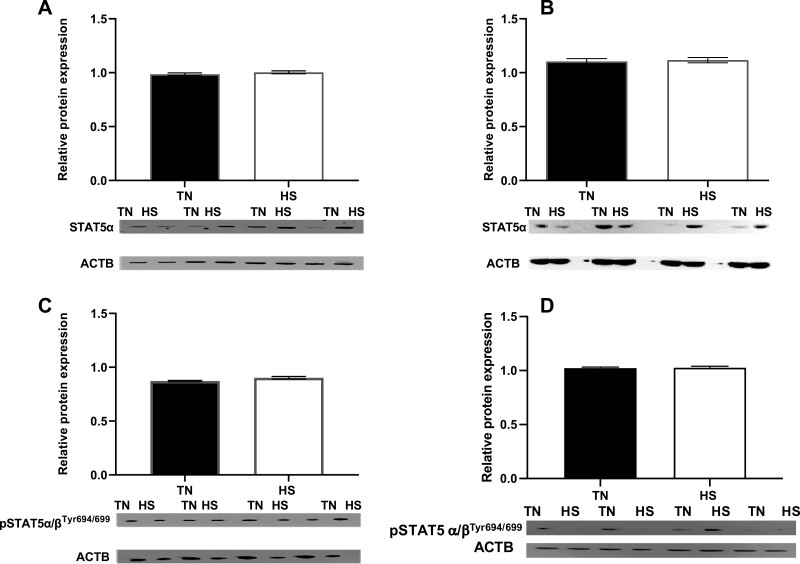
Heat stress effect on STAT5α and pSTAT5α/β^Tyr694/699^. (A, B) Gilts were exposed to TN (20 ± 1.2 °C) or cyclic HS (25.4 to 32 ± 1.2 °C) during the follicular phase for 5 d. (C, D) Gilts were exposed to TN (20 ± 2.6 °C) or diurnal HS (32 to 35 ± 2.6 °C) during the luteal phase from 3 to 12 dpe. Western blots with positive staining for STAT5α or pSTAT5α/β^Tyr694/699^ or ACTB proteins are presented. The abundance of (A, C) STAT5α:ACTB and (B, D) pSTAT5α/β^Tyr694/699^:ACTB protein ± SEM is presented; ^†^*P* < 0.10. Abbreviations: ACTB, beta-actin; dpe, days postestrus; HS, heat stress; pSTAT5α/β^Tyr694/699^, phosphorylated signal transducer and activator of transcription α/β tyrosine 694/699; STAT5α, signal transducer and activator of transcription 5α; TN, thermoneutral.

No effect of HS on ovarian (FOL) or luteal (LUT) STAT5α protein abundance was observed (*P* = 0.34; *P* = 0.75, respectively; Figure 3A and C). However, ovarian pSTAT5α/β^Tyr694/699^ tended to be increased (*P* = 0.07; Figure 3B) in FOL HS-exposed gilt ovaries, although unaltered (*P* = 0.76; Figure 3D) in luteal tissue in LUT gilts.

### Impact of HS on STAT1 protein abundance and phosphorylation

STAT1, pSTAT1^Ser727^, and pSTAT1^Tyr701^ proteins were not detectable in ovaries or CL from FOL and LUT gilts, respectively (data not shown).

### Effect of HS on the ratio of phosphorylated JAK-STAT proteins

**Figure 4.**
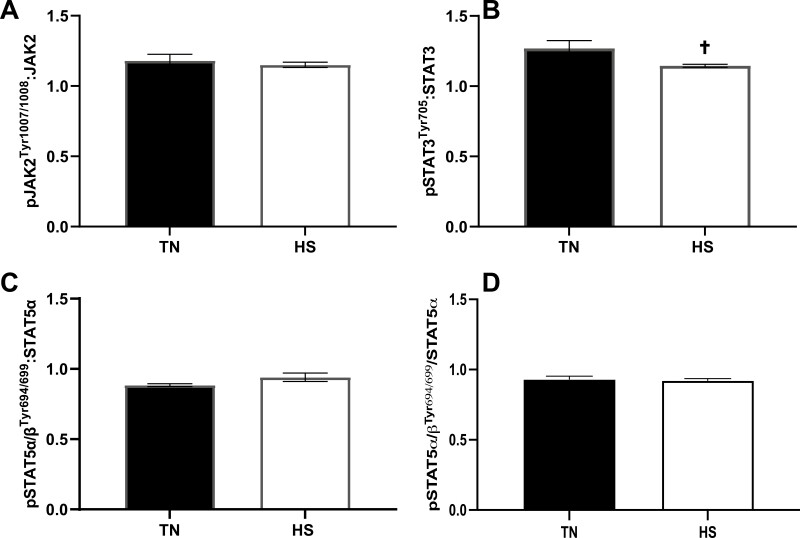
Effect of heat stress on the ratio of phosphorylated JAK-STAT pathway proteins. The relative ratio of (A) pJAK2^Tyr1007/1008^:JAK2, (B) pSTAT3^Tyr705^:STAT3, (C) pSTAT5α/β^Tyr694/699^:STAT5α during the luteal phase and (D) pSTAT5α/β^Tyr694/699^:STAT5α during the follicular phase ± SEM is presented; ^†^*P* < 0.10. Abbreviations: pJAK2^Tyr1007/1008^, phosphorylated janus kinase 2 tyrosine 1007/1008; pSTAT3^Tyr705^, phosphorylated signal transducer and activator of transcription 3 tyrosine 705; pSTAT5α/β^Tyr694/699^, phosphorylated signal transducer and activator of transcription α/β tyrosine 694/699; STAT3, signal transducer and activator of transcription 3; STAT5α, signal transducer and activator of transcription 5α.

The ratio of luteal pJAK2^Tyr1007/1008^ to total JAK2 was unaltered in LUT gilts (*P* = 0.57; Figure 4A) during HS. In contrast, luteal pSTAT3^Tyr705^ to total STAT3 tended to be decreased (*P* = 0.10; Figure 4B) in luteal tissue during HS. No effect of HS on ovarian (FOL) or luteal (LUT) pSTAT5α/β^Tyr694/699^ to total STAT5α ratio (*P* = 0.15; *P* = 0.75, respectively; Figure 4C and D) was observed.

## Discussion

In pigs, HS represents an animal welfare and economic concern and arises when a thermal energy imbalance occurs due to environmental conditions coupled with endogenous heat production (Bernabucci et al., 2010). Phenotypically, HS-induced SI manifests as increased spontaneous abortion (Love, 1978), delayed puberty (Paterson et al., 1991), lowered conception rate, and reduced embryonic viability (Omtvedt et al., 1971). Swine have insufficient functional sweat glands to dissipate accumulated heat and they respond to elevated core temperature by initiating peripheral vasodilation (Ingram, 1967; Alamer, 2011) and increasing respiration (Collier and Gebremedhin, 2015). Redirection of blood to the skin reduces cardiovascular output to the splanchnic bed (Collin et al., 2001) and since the alimentary canal epithelium is sensitive to hypoxia (Wells et al., 1996; Hall et al., 1999) these HS-induced circulatory changes cause intestinal hyperpermeability (Kregel et al., 1988; Dokladny et al., 2006; Lambert, 2009; Pearce et al., 2012, 2013), resulting in LPS and other intestinal antigens infiltrating into local and systemic circulation (Pearce et al., 2013). In pituitary explants from ewes, LPS and gonadotrophin-releasing hormone (**GnRH**) costimulate PRL gene expression and secretion (Tomaszewska-Zaremba et al., 2018). Additionally, PRL regulates proliferation of T and B cells, macrophages, and interferon regulatory factor-1 (Nielsen et al., 1992; Wang et al., 1997; Yu-Lee, 2002), suggesting that immunological activation may contribute to elevated PRL secretion during hyperthermia, presumably due to HS-induced damage to the intestinal barrier.

Pancreatic insulin secretion is elevated in response to PRL (Sorenson et al., 1987), and gilts are hyperinsulinemic during HS, compared to thermal neutral pigs on a similar plane of nutrition (Baumgard and Rhoads, 2013; Sanz Fernandez et al., 2015; Kvidera et al., 2017). Porcine ovarian INSR, insulin receptor substrate 1 (**IRS1**), and protein kinase B (**AKT1**) mRNA levels increase during HS, along with increased phosphorylation of IRS1 and AKT1 (Nteeba et al., 2015), suggesting hyperactivation of ovarian insulin-mediated signaling. Several studies support a reproductive role for PRL including stimulation of 17β-estradiol (**E**_**2**_) and progesterone (**P**_**4**_) by PRL in gilts (McNeilly et al., 1982). Chronic hyperprolactinemia in rats reduced the level of gonadotrophin-induced E_2_ production in antral follicles (Jonassen et al., 1991), and hyperprolactinemia in women inhibits GnRH contributing to altered cyclicity, ovulatory failure, and infertility (Bachelot and Binart, 2007). Further, Prl null female mice are infertile with irregular estrous cycles (Horseman et al., 1997; Ormandy et al., 1997). Thus, PRL has essential roles in both insulin homeostasis and ovarian function. These physiological actions coupled with HS-induced hyperprolactinemia in multiple species (Baumgard and Rhoads, 2013) provided the rationale for this study.

Although HS increases circulating PRL in several species, including rodents (An et al., 2020), sheep (Schillo et al., 1978; Hooley et al., 1979), cattle (Alamer, 2011), and swine (Zhang et al., 2019; Li et al., 2021), in this study, there was no difference in circulating plasma PRL in either FOL or LUT gilt HS models. Secretion of PRL can be influenced by the level of E_2_ (Sosa et al., 2012) and HS exposure during both FOL and LUT gilt models were during periods of low but rising (FOL) or low (LUT) E_2_, suggesting increased circulating PRL may depend upon E_2_ production associated with estrous cyclicity, although HS in males also increases circulating PRL (Vesic et al., 2021). Gilts experienced TN treatments in the LUT model until 2 d postovulation to ensure completion of ovulation and luteinization before HS, which continued until peak luteal function on day 12. No effect on circulating P_4_ was observed despite the CL being smaller in size due to HS (Bidne et al., 2019). High levels of P_4_ suppress PRL levels (Chen and Meites, 1970); thus, the absence of an effect on circulating P_4_ during HS may contribute to the lack of an observed effect on plasma PRL during HS in the luteal phase. A previous study had noted increased circulating PRL after 1 and 7 d of HS (Sanz-Fernandez et al., 2012), while another study in pregnant sows determined decreased PRL after ~30 d of HS (Li et al., 2021). Although surprising, the length of HS may have been insufficient to observe hyperprolactinemia in both reproductive phases since gilts were exposed to acute HS exposures. Additionally, the study determining hyperprolactinemia after 1 and 7 d of HS was conducted in younger growing pigs (Sanz-Fernandez et al., 2012), while reduced PRL was identified due to HS in late gestation sows (Li et al., 2021); thus, age and developmental status of the female could be another potential contributing factor. The effects of HS on PRL therefore appear to be multifactorial and understanding how environment affects this important and pleiotropic hormone warrants further investigation.

Due to ascribed roles for PRL in thermotolerance and reproduction, examination of downstream pathway proteins mediated by PRL binding to its receptor was considered appropriate. PRLR is present in many peripheral tissues, and PRLR-deficient animal models have abnormal cyclicity, increased luteal cell apoptosis, reduced ovulation, and impaired P_4_ production (Grosdemouge et al., 2003). A single transmembrane cytokine receptor protein, PRLR exists as long (**PRLR-L**) and short (**PRLR-S**) form splice isoforms and vary among species (Kelly et al., 1991). These isoforms share a common extracellular domain but differ in their length of intracellular domains created by alternative splicing at the 3ʹ end of the PRLR gene (Kelly et al., 1992). The signaling transduction of downstream proteins is more limited with the PRLR-S compared to PRLR-L isoform and PRLR-S can exist as a homodimer or heterodimer with PRLR-L (Ormandy et al., 1998). In rats, PRLR-S inhibits PRLR-L-mediated activation of JAK2 kinase and downstream gene transcription (Chang and Clevenger, 1996; Bole-Feysot et al., 1998). In Senepol crossbred cattle, PRLR mutation induces a slick-type coat optimizing the ability to remain euthermic (Littlejohn et al., 2014). This study evaluated the ovarian abundance of both PRLR forms combined, and PRLR was detectable in both hyperthermic FOL ovaries and LUT CLs with no observable difference in protein abundance due to HS, implicating that while the ovarian PRLR is present, thermal stress did not alter it in this paradigm. Induction of porcine ovarian PRLR during the follicular phase suggests PRL may participate in follicle development, although a direct role remains unknown. Increased luteal PRLR was anticipated as PRLR protein expression occurs in the early luteal phase in domesticated gilts (Slomczynska et al., 2001). Similar observations in cattle of increased mRNA encoding PRLR-L and PRLR-S during late CL (days 15 to 17) relative to early, mid, and regressed luteal stages are documented (Shibaya et al., 2006).

PRL elicits its effects through the JAK/STAT pathway by activating and dimerizing the PRLR with further activation and autophosphorylation of JAK2. JAK2 activates STAT 1, 3, and 5 proteins, resulting in STAT1, STAT3, and STAT5 dimerization and nuclear translocation necessary for the cellular response (Bole-Feysot et al., 1998; Farmer and Palin, 2005). In this study, total and phosphorylated ovarian JAK2 protein abundance was undetectable in FOL gilt ovaries, but LUT gilt ovaries had measurable luteal JAK2 and pJAK2^Tyr1007/1008^. Thus, autophosphorylation of JAK2 may differ dependent upon the stage of the estrous cycle in gilts. In COV434 human granulosa carcinoma cells, JAK2 abundance is low and not impacted by administration of a Janus kinase 1 (**JAK1**)/JAK2 inhibitor relative to JAK1 (Frost et al., 2020), implying that JAK2 may not be the primary kinase activated during the follicular phase and may potentially depend upon the presence of luteal cells. Interestingly, there was no HS impact on luteal JAK2 or pJAK2^Tyr1007/1008^ abundance in LUT gilt ovaries. This was unexpected since febrile exposure downregulates JAK2 protein abundance (Nespital and Strous, 2012), and various cytokine receptors stabilize JAK2 (Ihle, 1995; Parganas et al., 1998). Exposure of porcine skeletal muscle to HS for up to 6 h increased the abundance of JAK2 while decreasing STAT3 and pSTAT3^Tyr705^ (Ganesan et al., 2017), suggesting a tissue-specific and/or time-dependent response of JAK-STAT signaling during hyperthermia. Thus, ovarian signal transduction activation of JAK2 cannot be solely attributed to PRL-ligand binding and could contribute to the lack of an observable effect in this study.

The STAT1 protein regulates apoptotic genes during ischemia and genotoxic stress (Stephanou and Latchman, 2003). Heat shock of human Jurkat cells elevated total STAT1 and pSTAT1^Tyr701^ (Chen et al., 2007). In nonovarian human cell line, treatment by interferon-γ elevated heat shock protein 70 and 90 during STAT1 activation, with no detection observed in STAT1-deficient cells under normal environmental conditions (Stephanou et al., 1999). Also, cultured porcine granulosa cells transfected with STAT1 increased the abundance of proapoptotic caspase-3 and bcl-2 associated X protein but not proliferative cell nuclear antigen, suggesting STAT1 potentially contributes to reduced follicle growth and atresia (Benco et al., 2009). Thus, these findings indicate a role for STAT1 in fertility and responsiveness to thermal stress. In our study, STAT1 protein was not detected in ovarian or luteal tissue from FOL and LUT gilts, respectively. This is supported in other studies in which the abundance of STAT1 was unaltered in vitro in follicle-stimulating hormone-treated cultured mouse granulosa cells with gradual reduction of pSTAT1^Ser727^ and no stimulation of pSTAT1^Tyr701^ after 6, 12, and 24 h of treatment (Du et al., 2015). Luteal STAT1 mRNA in pregnant cows was elevated 3-fold compared to cyclic CL on day 18 of estrous, independent of P_4_ (Basavaraja et al., 2019), and luteal STAT1 was undetectable in Wistar rats; however, supplementation with luteinizing hormone (**LH**) elevated STAT1 (Carvalho et al., 2003). Thus, STAT1 abundance in LUT gilts may depend upon LH secretion as in this study the gilts experienced HS 2 d postovulation. Additionally, weak STAT1 abundance in the oocyte and granulosa cells of aging ovaries suggests that STAT1 may not be a major ovarian STAT protein (Frost et al., 2020) further supporting the lack of STAT1 detection in the early follicular phase.

Immature and atretic granulosa cells express STAT3, which is absent in preovulatory granulosa cells (Russell and Richards, 1999). Mild heat shock reduced heat shock protein abundance in the absence of STAT3 in vitro and in *Stat3* knockout mice, suggesting STAT3 activation is essential for thermotolerance (Matozaki et al., 2019). Porcine STAT3 mRNA and protein are abundant in reproductive relative to other tissues (Wen et al., 2006), and STAT3 and pSTAT3 are increased in gilt granulosa cells by epidermal growth factor stimulation (Wen et al., 2006). In our study, FOL ovarian STAT3 abundance was unaltered by HS, and pSTAT3^Tyr705^ was not detectable, indicating the presence of STAT3 during the follicular phase, albeit without any influence of HS. Luteal abundance of total STAT3 was increased by HS on 12 dpe, suggesting HS alters luteal STAT3 as a mode of action. However, luteal pSTAT3^Tyr705^ was unaltered by HS, though pSTAT3^Tyr705^:STAT3 ratio was reduced. Since CL diameter and weight were reduced by HS (Bidne et al., 2019), pSTAT3^Tyr705^ might have a role in HS-reduced luteal size due to a cell growth or viability function.

Initially, STAT5 was identified as the main STAT family transcription factor activated by PRLR (Ali and Ali, 1998). *Stat5α/β*-deficient mice have reduced numbers of large CL affecting fecundity (Teglund et al., 1998). Adult Wistar rats exposed to a bolus of insulin during diestrus had elevated phosphorylation of STAT5β in theca and granulosa cells (Carvalho et al., 2003). We did not observe a difference in FOL ovarian STAT5β abundance, but HS increased pSTAT5α/β^Tyr694/699^, suggesting activation of pSTAT5α/β^Tyr694/699^ during the follicular phase in gilt ovaries. No HS impact was observed in gilts during the luteal phase, demonstrating differential impacts of HS on pSTAT5α/β^Tyr694/699^ dependent on the phase of estrous cyclicity.

To summarize, a plethora of physiological functions of PRL have been identified (Bole-Feysot et al., 1998) related to pancreatic beta-cell growth (Nielsen et al., 1992), insulin release (Sorenson et al., 1987; Petryk et al., 2000), and thermoregulation (Littlejohn et al., 2014). Despite this, HS did not increase circulating PRL during either the follicular or luteal phase of the estrous cycle in postpubertal gilts. There was also no impact of HS on the abundance of ovarian PRLR, JAK2, or pJAK2^Tyr1007/1008^ in either stage. During the follicular phase, HS increased pSTAT5α/β^Tyr694/699^. During the luteal stage, STAT3 was increased and the ratio of pSTAT3^Tyr705^:STAT3 was concomitantly decreased by HS. Thus, these data suggest that the JAK/STAT signaling pathway is moderately altered by HS during different estrous cycle stages, but these appear independent of PRL.

## Abbreviations

ACTB: beta-actin
AKT1: protein kinase B
CL: corpus luteum
dpe: days postestrus
E_2_: 17β-estradiol
FOL: follicular phase
GnRH: gonadotrophin-releasing hormone
HRP: horseradish peroxidase
HS: heat stress
IgG: immunoglobulin G
INSR: insulin receptor
IRS1: insulin receptor substrate 1
JAK1: Janus kinase 1
JAK2: Janus kinase 2
JAK-STAT: Janus kinase/signal transducer and activator of transcription
LH: luteinizing hormone
LPS: lipopolysaccharide
LUT: luteal phase
mRNA: messenger RNA
P_4_: progesterone
PBS: phosphate-buffered saline
PBST: phosphate-buffered saline with Tween-20
pJAK2^Tyr1007/1008^: phosphorylated janus kinase 2 tyrosine 1007/1008
PRL: prolactin
PRLR: prolactin receptor
PRLR-L: prolactin receptor-long
PRLR-S: prolactin receptor-short
pSTAT1^Ser727^: phosphorylated signal transducer and activator of transcription 1 serine 727
pSTAT1^Tyr701^: phosphorylated signal transducer and activator of transcription 1 tyrosine 701
pSTAT3^Tyr705^: phosphorylated signal transducer and activator of transcription 3 tyrosine 705
pSTAT5α/β^Tyr694/699^: phosphorylated signal transducer and activator of transcription α/β tyrosine 694/699
SI: seasonal infertility
STAT1: signal transducer and activator of transcription 1
STAT3: signal transducer and activator of transcription 3
STAT5α: signal transducer and activator of transcription 5α
TN: thermoneutral
